# Development of a Droplet Digital Polymerase Chain Reaction for Sensitive Detection of *Pneumocystis jirovecii* in Respiratory Tract Specimens

**DOI:** 10.3389/fmed.2021.761788

**Published:** 2021-12-22

**Authors:** Jie Yi, Nan Wang, Jie Wu, Yueming Tang, Jingjia Zhang, Lingxiang Zhu, Xiao Rui, Yong Guo, Yingchun Xu

**Affiliations:** ^1^State Key Laboratory of Complex Severe and Rare Diseases, Peking Union Medical College Hospital, Chinese Academy of Medical Sciences, Beijing, China; ^2^Beijing Key Laboratory for Mechanisms Research and Precision Diagnosis of Invasive Fungal Diseases, Beijing, China; ^3^Department of Clinical Laboratory, Peking Union Medical College Hospital, Chinese Academy of Medical Sciences, Beijing, China; ^4^Human Genetic Resource Center, National Research Institute for Family Planning, Beijing, China; ^5^Graduate School of Peking Union Medical College, Chinese Academy of Medical Sciences, Beijing, China; ^6^Department of Biomedical Engineering, School of Medicine, Tsinghua University, Beijing, China; ^7^TargetingOne Corporation, Beijing, China

**Keywords:** *Pneumocystis jirovecii*, DNA, quantitative PCR, droplet digital PCR, sensitivity

## Abstract

**Background:** *Pneumocystis jirovecii* is a human-specific opportunistic fungus that causes Pneumocystis pneumonia (PCP), a life-threatening opportunistic lung infection that affects immunocompromised patients. *P. jirovecii* colonization may be linked to the transmission of the infection. The detection of *P. jirovecii* in immunocompromised patients is thus especially important. The low fungal load and the presence of PCR inhibitors limit the usefulness of quantitative PCR (qPCR) for accurate absolute quantification of *P. jirovecii* in specimens. Droplet digital PCR (ddPCR), however, presents a methodology that allows higher sensitivity and accuracy. Here, we developed a ddPCR method for detecting *P. jirovecii* DNA in respiratory specimens, and evaluated its sensitivity against qPCR.

**Materials and Methods:** One bronchoalveolar fluid (BALF) sample each was collected from 82 patients with potential PCP to test the presence of *P. jirovecii* DNA using both ddPCR and qPCR, and samples with inconsistent results between the two methods were further tested by metagenomic next generation sequencing (mNGS). In addition, 37 sputum samples from 16 patients diagnosed with PCP, as well as continuous respiratory tract specimens from nine patients with PCP and treated with sulfonamides, were also collected for *P. jirovecii* DNA testing using both ddPCR and qPCR.

**Results:** ddPCR and qPCR gave the same results for 95.12% (78/82) of the BALF samples. The remaining four specimens tested positive using ddPCR but negative using qPCR, and they were found to be positive by mNGS. Detection results of 78.37% (29/37) sputum samples were consistent between ddPCR and qPCR, while the other eight samples tested positive using ddPCR but negative using qPCR. The *P. jirovecii* load of patients with PCP decreased to undetectable levels after treatment according to qPCR, but *P. jirovecii* was still detectable using ddPCR.

**Conclusions:** ddPCR was more sensitive than qPCR, especially at detecting low-pathogen-load *P. jirovecii*. Thus, ddPCR represents a useful, viable, and reliable alternative to qPCR in *P. jirovecii* testing in patients with immunodeficiency.

## Introduction

*Pneumocystis jirovecii* is a human-specific opportunistic fungus that causes Pneumocystis pneumonia (PCP), a life-threating lung infection that affects immunocompromised individuals (1) and is a common opportunistic infection in *Human immunodeficiency virus* (HIV)-infected patients with CD4 counts <200 cells/mm^3^ (2). Patients presenting with PCP may show signs of fever, cough, dyspnea, and, in severe cases, respiratory failure (3, 4). Not only *P. jirovecii* colonization in patients may be linked to the transmission of the infection, but also it may be linked to the development or transmission of PCP (5). Therefore, early detection of *P. jirovecii* in immunocompromised patients has become particularly important. In general, *P. jirovecii* is detected in respiratory specimens through microscopic examination followed by staining, which has low sensitivity and specificity (6). Detection of serum β-D-glucan is sensitive but lacks specificity (7). Polymerase chain reaction (PCR) has been used for the confirmation of microscopically positive PCP (8). However, there have been no commercial PCR kits approved for clinical use in China and the sensitivity of the PCR method is not satisfactory (9).

Droplet digital PCR (ddPCR), as a new and accurate quantitative technique, is an absolute quantitative method for the detection of genes (10, 11), especially for trace specimens (12–14). Compared with quantitative PCR (qPCR), ddPCR has higher sensitivity and precision, so it can be used for early detection and subsequent monitoring of treatment of low-load pathogenic microorganisms, providing quantitative thresholds for judgment (15–18). ddPCR has been successfully used to detect *Mycobacterium tuberculosis* in plasma specimens of pulmonary tuberculosis patients (19). Strain et al. demonstrated that the high sensitivity and precision of ddPCR can help to measure the level of latent HIV so that intervention can be given in time to achieve early elimination of the virus (20). Our group has developed a ddPCR assay for high-sensitivity detection of SARS-Cov-2 (14, 21, 22). In addition, the high sensitivity of ddPCR is shown to be suitable for the detection of microviruses in aqueous specimens (14, 15, 21, 22).

In this study, we developed a ddPCR method to detect *P. jirovecii* in respiratory specimens, and evaluated its sensitivity (as compared with qPCR), in order to provide an effective and sensitive method for detecting *P. jirovecii* in immunocompromised patients.

## Materials and Methods

### Design of Primers and Probes

The primers and probes were designed using the NCBI primer-blast software. The sequences used were the mtLSU reference sequence of *P. jirovecii* (accession number NC_020331.1) and the human reference gene N-acetylglucosamine kinase (*NAGK*, accession number NC_000002.12). The primers and probes were evaluated for specificity by NCBI blast and the sequence structures (for example, hairpin or self-dimer structure) were assessed by Oligo7 software (Cascade, CO, USA). The Vector NTI Advance software (Invitrogen, Carlsbad, CA, USA) was employed to evaluate the mtLSU sequences of multiple *P. jirovecii* isolates, to avoid designing primers and probes in regions with high-frequency single nucleotide polymorphisms. All primers and probes were synthesized by Sangon Biotech Ltd. (Shanghai, China). The sequences of the primer–probe sets are shown in Table 1.

**Table 1:** Target genes and primer–probe set sequences for *P. jirovecii* DNA detection.

**Target**		**Primer–probe set**
Mitochondrial large	Forward primer	GTATAGCACTGAATATCTCGAGGG
subunit rRNA gene	Reverse primer	GAGCTTTAATTACTGTTCTGGGCT
(*P. jirovecii*)	Probe	FAM-TTCGACTATCTACCTTATCGC
		-MGB
N-acetylglucosamine	Forward primer	AGATGCTGGGCAGACACATC
kinase gene	Reverse primer	CCCACCTTCACTCCCACCT
(*Homo sapiens*)	Probe	HEX-AGCAGTGTTGCCCGAGATTG
		ACCC-BHQ1

### Study Participants

A retrospective study of 98 non-HIV immunocompromised patients with suspected PCP were recruited from Peking Union Medical College Hospital (PUMCH) between June 2019 and December 2020. The following clinical and biological data for each patient were recorded: underlying disease (hematological malignancies, autoimmune disease, lung diseases, cancer, and other diseases), radiological signs (obtained by X-ray analysis or computed tomography scan), data from a biological workup (results of direct physical examination), treatments [curative and prophylactic treatments, long-term treatment for the underlying disease (e.g., corticosteroids, chemotherapies, HAART)], and clinical outcome. Patients who had no laboratory test results, no chest radiograph, or whose medical records were unavailable were excluded. The bronchoalveolar fluid (BALF) or sputum specimens were collected more than once in the patients, but only the first qPCR result was evaluated in this study.

All specimens were isolated from patients as part of routine diagnosis and treatment, and no unnecessary invasive procedures were performed. This study was approved by the Ethics Committee of PUMCH (S-T767).

### Diagnosis of PCP

The following criteria were used for the diagnosis of PCP: (1) BALF or sputum GMS staining yielded *P. jirovecii*. (2) BALF or sputum qPCR yielded positive results in two replicates. A definitive diagnosis was defined as meeting criterion (1),and a clinical diagnosis was defined as meeting criterion (2). Patients meeting (1) and (2) criteria were classified as PCP cases, and patients meeting (1) or (2) criteria were classified as potential cases.

### Staining for *P. jirovecii*

A portion of resuspended pellet of sputum or BALF was used to make a smear for GMS staining, according to the manufacturer's instructions (Baso, Zhuhai, China). The stained smears were visualized under a microscope for Pneumocystis cysts.

### DNA Extraction

Spontaneously expectorated sputum from a deep cough and BALF were collected and examined (21, 23). The sputum or BALF was mixed with an equal volume of Sputasol (Thermo Fisher Scientific, Waltham, MA, U.S.A.) containing 0.1% dithiothreitol and incubated for 15 min at 37°C. The homogenized sputum or BALF (1 mL) was centrifuged (13,000 rpm for 10 min) and the pellet resuspended in 500 μL sterile PBS (GE Lifesciences, Marlborough, MA, U.S.A.). DNA extraction was performed using the Human Genome Nucleic Acid Extraction Kit (Tianlong, Xi'an, China) according to the manufacturer's instructions.

### ddPCR Detection of *P. jirovecii*

Detection of *P. jirovecii* by ddPCR was performed via the TargetingOne Digital PCR System (TargetingOne, Beijing, China) (24). The 30 μL PCR reaction mix included 7.5 μL 4× SuperMix, 3 μL 10× primers and probes mix (the final concentrations of primers and probes were 600 and 300 nM, respectively), and up to 15 μL of the DNA template. The PCR mix was thoroughly blended and added into the droplet generation chip. Then, 180 μL of droplet generation oil was added and the chip was put into the Drop Maker for droplet generation. The generated droplets were then automatically transferred into an 8-strip PCR tube and reacted on Thermal Cycler with conditions as follows: pre-denaturation at 95°C for 10 min; amplification for 45 cycles with denaturation at 94°C for 30 s and annealing at 57°C for 1 min; and ending at 12°C. Subsequently, the 8-strip PCR tube containing the amplified droplets was connected to a droplet detection chip and detected using the Chip Reader. Finally, the data were analyzed by the dedicated software to obtain the target DNA copy numbers. All procedures were following the manufacturer's instructions.

### qPCR Detection of *P. jirovecii*

The presence of *P. jirovecii* from human airway specimens was also assessed by a probe-based qPCR assay. The primers and probes were the same as those used in the ddPCR. Each TaqMan reaction mix contained 1× PCR master mix (Promega, Beijing, China), 900 nM of forward and reverse primers each, 250 nM of probe, 15 μL of DNA template to make a final volume of 50 μL. Reactions were performed with the Applied Biosystems® Real-Time PCR System (Applied Biosystems, Foster City, CA, U.S.A.) under the following conditions: 95°C for 5 min, followed by 40 cycles of denaturation at 95°C for 3 s and annealing/extension at 60°C for 30 s. The result was considered valid only when the cycle threshold (Ct) value of the reference gene was ≤ 40. The result was considered positive when the Ct values of both target genes were ≤ 37, negative when they were both > 37.

### Metagenomic Next Generation Sequencing and Data Analysis

BALF specimens from patients were collected according to standard operating procedures. For DNA extraction, 1.5 mL microcentrifuge tubes each containing a 0.6 mL sample and 1 g 0.5 mm glass beads were attached to a horizontal platform on a vortex mixer and agitated vigorously at 2,800–3,200 rpm for 30 min. Then 0.3 mL of the sample was separated into a new microcentrifuge tube, and the total DNA was extracted using the TIANamp Micro DNA Kit (Tiangen, Beijing, China) according to the manufacturer's recommendation. The total DNA was subjected to library construction through DNA-fragmentation (150 bp), end-repair, adapter-ligation, and unbiased PCR amplification. Agilent 2100 was used for quality control of the DNA libraries (200–300 bp). Qualified libraries were sequenced by BGISEQ-50 platform (25). After removing low-quality reads (<35 bp) and computationally subtracting sequences mapped to the human reference genome (hg19) from the sequencing data by Burrows-Wheeler Alignment (0.7.10-r789) (26), high-quality sequences were generated. Following the removal of low-complexity reads according to Prinseq (version 0.20.4), the remaining sequences were phylogenetically classified by aligning to PMDB (PMseq metagenomic Database, version 3.0), a BGI-locally established database consisting of 2,700 whole genome sequences of viral taxa, 1,494 bacterial genomes or scaffolds, 73 fungi related to human infection, and 47 parasites associated with human diseases, which were downloaded from NCBI (ftp://ftp.ncbi.nlm.nih.gov/genomes/).

### Statistical Analysis

Comparisons between two groups were made using the Mann Whitney *U* test. The correlation between the Ct values of qPCR and *P. jirovecii* DNA copy number determined by ddPCR was analyzed with the Spearman correlation test. A *P* < 0.05 (two-sided) was considered statistically significant. The above-mentioned analyses were performed using either GraphPad Prism 7.0 (La Jolla, CA, U.S.A.) or SPSS 19.0 (College Station, TX, U.S.A.) software.

## Results

### Demographic and Clinical Features of Study Participants

The present study included 98 patients including diagnosed PCP and potential PCP patients. The characteristics of the patients are shown in Table 2. For diagnosed PCP patients, the median age was 59 years (range, 22–67 years) and the proportion of men was 81.13%; the most common underlying diseases were lung diseases (26.42%) and autoimmune diseases (24.52%), and other diseases included hematological malignancies (13.20%), cancers (13.20%), and others (22.64%). Whereas for potential PCP patients, the median age was 56 years (range, 19–80 years) and the proportion of men was 48.89%; the most common underlying diseases were others (37.78%) and lung diseases (24.44%), followed by cancers (15.56%), autoimmune diseases (11.11%), and hematological malignancies (11.11%).

**Table 2:** Patient characteristics.

**Characteristic**	**Diagnosed PCP**	**Potential PCP**	***P*** **value**
	**patients (***n*** = 53)**	**patients (***n*** = 45)**	
Age (years), median (range)	59 (22–67)	56 (19–80)	0.37
Male, *n* (%)	43 (81.13)	22 (48.89)	1.00
**Underlying disease**, ***n*** **(%)**			
Autoimmune diseases	13 (24.52)	5 (11.11)	1.00
Hematological malignancies	7 (13.20)	5 (11.11)	1.00
Lung diseases	14 (26.42)	11 (24.44)	1.00
Cancers	7 (13.20)	7 (15.56)	1.00
Others	12 (22.64)	17 (37.78)	1.00

*PCP, Pneumocystis jirovecii pneumonia*.

### Assessment of the Specificity of the *P. jirovecii* TaqMan Primer and Probe Set by ddPCR

Several DNA samples of airway specimens from patients with respiratory infections other than PCP were used to assess the specificity of the *P. jirovecii* TaqMan primer and probe set. All these DNA samples were previously validated by metagenomic next generation sequencing (mNGS). The pathogens of these specimens included bacteria *Pseudomonas aeruginosa, Staphylococcus aureus, Klebsiella pneumoniae, Acinetobacter baumannii, Enterococcus faecalis*, and *mycoplasma*; fungi *Candida albicans, Candida parapsilosis*, and *Cryptococcus neoformans*, as well as respiratory viruses *herpes simplex virus-1* (HSV-1) and *parainfluenza virus*. As shown in Table 3, when ddPCR was used for testing, no nonspecific amplification of DNA of other pathogens was found using this primer and probe set for *P. jirovecii*.

**Table 3:** Specificity results of *P. jirovecii* TaqMan primer and probe set.

**Specimen**	**Sample type**	**Pathogens identified by mNGS**	**Pathogens identified by *P. jirovecii* ddPCR**
1	BALF	*Pseudomonas aeruginosa, Staphylococcus aureus*	N
2	Sputum	*Mycoplasma*	N
3	BALF	*Klebsiella pneumoniae, Acinetobacter baumannii*	N
4	BALF	*Enterococcus faecalis*	N
5	BALF	*Candida parapsilosis, Cryptococcus neoformans*	N
6	Sputum	*Candida albicans*	N
7	BALF	*Parainfluenza virus*	N
8	Sputum	HSV-1	N

*ddPCR, droplet digital PCR; qPCR, quantitative PCR; P. jirovecii, Pneumocystis jirovecii; BALF, bronchoalveolar fluid; HSV-1, herpes simplex virus type-1. N, Negative*.

### Evaluation of the Limit of Blank, Limit of Detection, and Linearity Range of the ddPCR-Based Detection of *P. jirovecii*

In order to assess the limit of blank (LoB) and limit of detection (LoD), we used a human cDNA sample as a blank control template in 60 repeated experiments and calculated the LoB and LoD according to the formula in Table 4 (27). The results showed that the LoB of *P. jirovecii* DNA is 0 and the LoD is 3 copies/test. We then used the same human cDNA sample as template for another 30 experiments to validate the LoD.

**Table 4:** Calculation of LoB and LoD from *A*_FP_ counts.

**AFP**	**LoB (copies/test)**	**LoD (copies/test)**
0	0	3
0–0.05	1	5
>0.05	A_FP_ + 1.645 $\sqrt{{\text{A}}_{\text{FP}}}$ + 0.8	(1.645 + $\sqrt{1.645^{2}+4\text{LoB}}$)^2^/4

*LoB, limit of blank; LoD, limit of detection*.

*A_FP_, Average false positive*.

**Figure 1:**
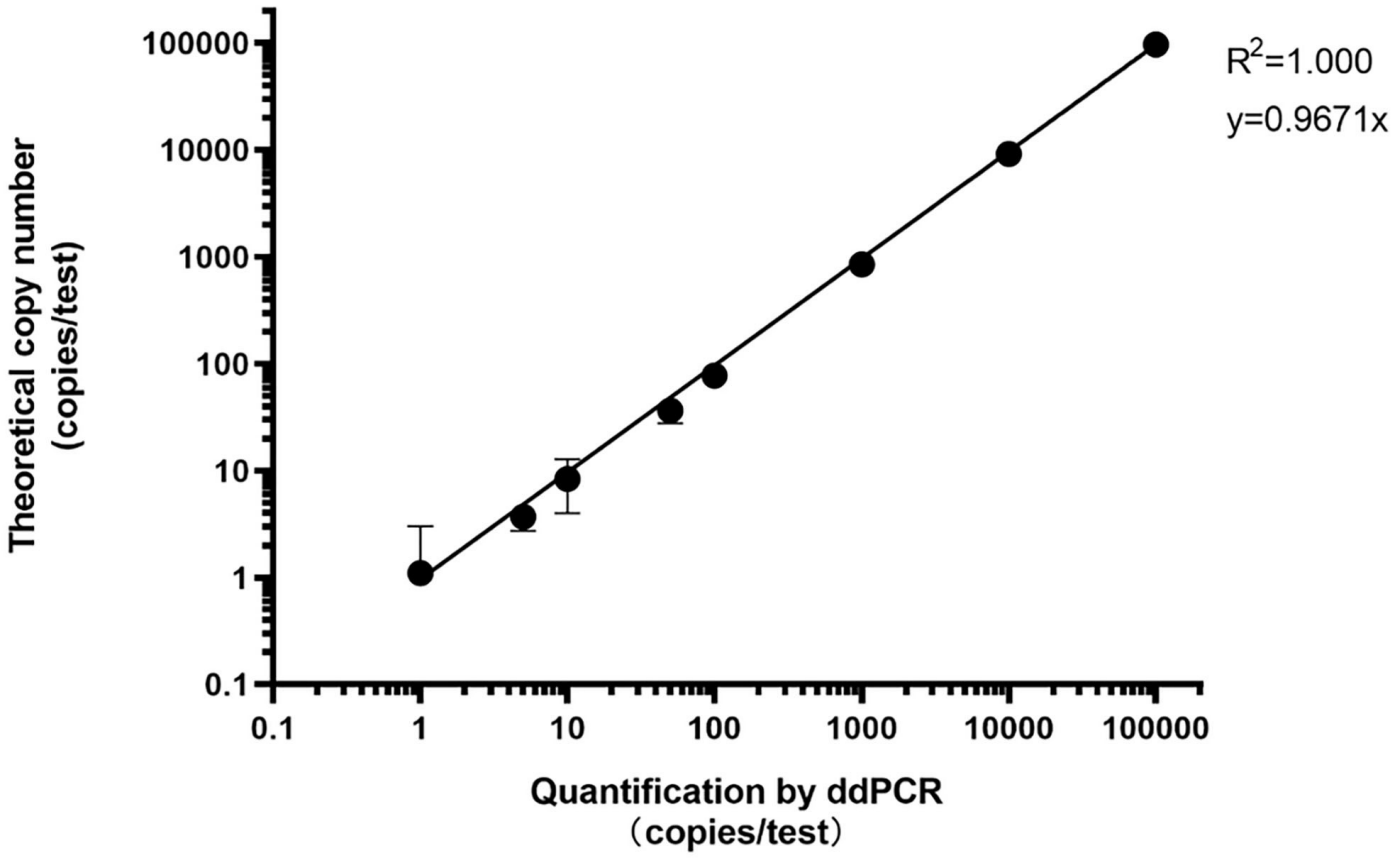
Dynamic range of *P. jirovecii* DNA detection using ddPCR.

We used a plasmid containing *P. jirovecii* mtLSU as the template to determine the linear range. First, the plasmid copy number was calculated based on plasmid concentration and sequence using the DNA/RNA copy number calculator (http://endmemo.com/bio/dnacopynum.php). ddPCR was performed using 10-fold serially diluted *P. jirovecii* mtLSU plasmids from 10^5^ copies/test to 1 copy/test, and each concentration was tested in triplicate. The results showed that the slope was 0.9671 (Figure 1).

### Performance of ddPCR in BALF Testing Compared With qPCR

**Figure 2:**
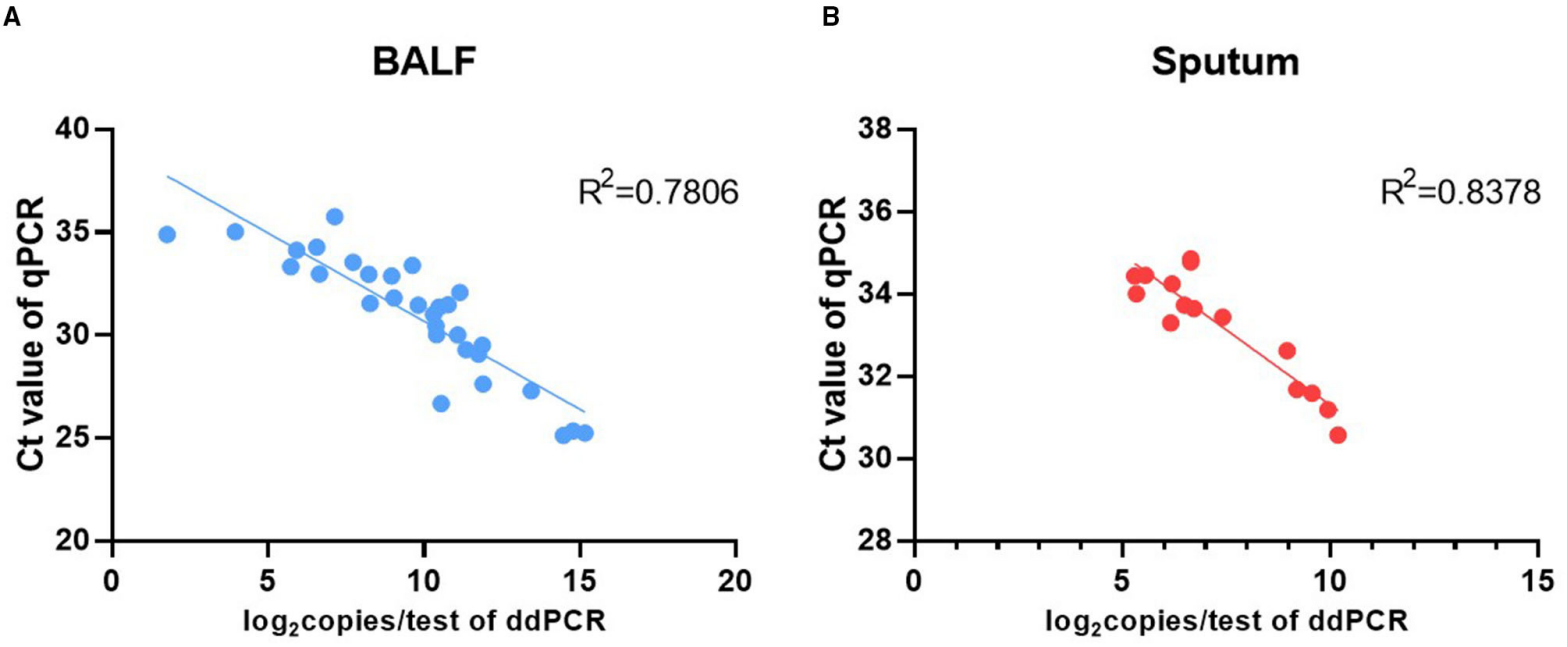
Correlation analysis between the Ct value of qPCR and the *P. jirovecii* load of ddPCR in BALF **(A)** and sputum **(B)**.

BALF specimens from 82 patients were analyzed using ddPCR and qPCR. The results showed that 43.90% (36/82) specimens were positive and 56.10% (46/82) negative using ddPCR, while 39.02% (32/82) specimens were positive and 60.96% (50/82) negative using qPCR. The Ct value of qPCR was highly correlated with the copy number determined by ddPCR (R^2^ = 0.7806) (Figure 2A). The results for 95.12% (78/82) of the samples were coincident between the two methods but the results of the remaining four specimens were inconsistent. The results of ddPCR for those four specimens were 5.60 copies/test, 4.40 copies/test, 9.00 copies/test, and 3.90 copies/test, while the results of qPCR for them were all negative. These four specimens were further tested by mNGS and the results were positive (Table 5).

**Table 5:** ddPCR, qPCR, and mNGS results of BALF specimens showing inconsistent results between ddPCR and qPCR.

**Specimen**	***P. jirovecii*** **DNA by ddPCR (copies/test)**	***P. jirovecii*** **DNA by qPCR (Ct value)**	**mNGS**
S1	5.60	Undetermined	*Pneumocystis jirovecii, Acinetobacter lwof*
S2	4.40	Undetermined	*Pneumocystis jirovecii*
S3	9.00	Undetermined	*Pneumocystis jirovecii, Acinetobacter baumannii, Ralstonia mannitolilytica, Pseudomonas aeruginosa, Achromobacter xylosoxidans, Streptococcus pneumoniae, Cytomegalovirus*
S4	3.90	Undetermined	*Pneumocystis jirovecii*

*ddPCR, droplet digital PCR; qPCR, quantitative PCR; P. jirovecii, Pneumocystis jirovecii; BALF, bronchoalveolar fluid; mNGS, metagenomic next generation sequencing; Ct, cycle threshold*.

### Performance of ddPCR in Sputum Testing Compared With qPCR

A total of 37 sputum specimens from 16 patients with diagnosed PCP were analyzed using ddPCR and qPCR. The results showed that 62.16% (23/37) specimens were positive and 37.84% (14/37) negative using ddPCR, while 40.54% (15/37) specimens were positive and 59.45% (22/37) negative using qPCR. The results of 78.37% (29/37) specimens were coincident between the two methods. The remaining eight specimens, including two groups of continuous specimens, were positive by ddPCR but negative by qPCR (Table 6). The Ct value of qPCR was highly correlated with the copy number determined by ddPCR (R^2^ = 0.84) (Figure 2B).

**Table 6:** Sputum specimens with inconsistent results by ddPCR and qPCR.

**Patient**	**Specimen**	***P. jirovecii*** **DNA by ddPCR (copies/test)**	***P. jirovecii*** **DNA by qPCR (Ct value)**
Patient 1	S1	40.10	Undetermined
Patient 2	S2	32.00	Undetermined
	S3	18.20	Undetermined
Patient 3	S4	28.90	Undetermined
Patient 4	S5	14.40	Undetermined
Patient 5	S6	14.90	Undetermined
	S7	4.50	Undetermined
Patient 6	S8	5.40	Undetermined

*ddPCR, droplet digital PCR; qPCR, quantitative PCR; P. jirovecii, Pneumocystis jirovecii; Ct, cycle threshold*.

### Comparison of *P. jirovecii* DNA Copy Number Tracking in Continuous Respiratory Tract Specimens by ddPCR and qPCR

**Figure 3:**
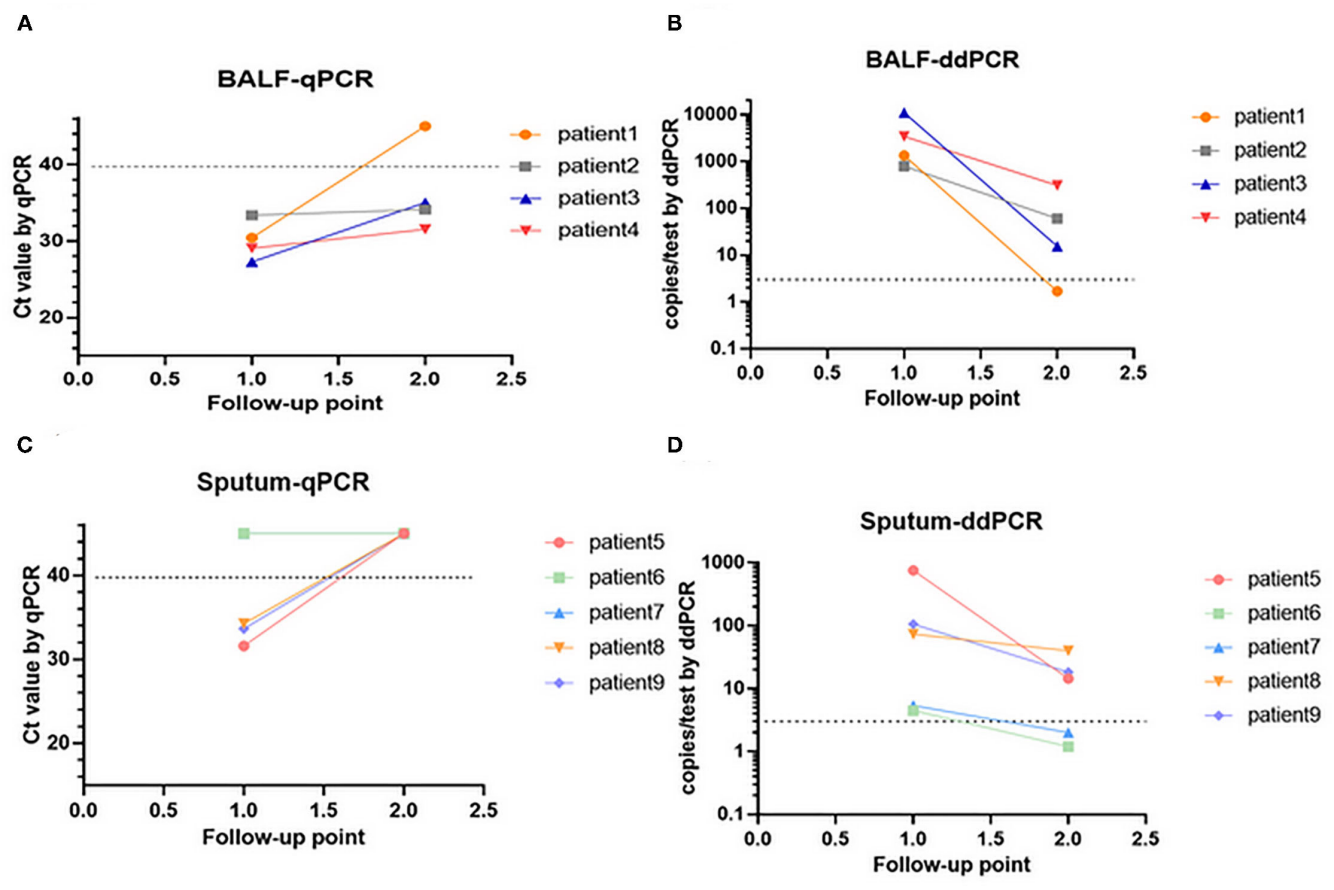
Changes in *P. jirovecii* load in continuous BALF specimens by ddPCR **(A,B)**. Changes in *P. jirovecii* load in continuous sputum specimens by qPCR **(C,D)**.

Continuous respiratory tract specimens from nine patients were collected. For patient 1 to patient 4, two continuously collected BALF specimens were collected from each patient; for patients 5 to patient 9, two continuous sputum specimens were collected from each patient. The sampling dates were shown in Supplementary Table 1. All patients were diagnosed with PCP and treated with sulfonamides. Specimens from all patients were collected before and during PCP treatment, and then analyzed using ddPCR (Figures 3A,C) and qPCR (Figures 3B,D). The *P. jirovecii* DNA copy number of these patients decreased after treatment according to qPCR and ddPCR. However, *P. jirovecii* DNAs of some specimens became undetectable (specifically, patients 1, 5, 7, 8, and 9) using qPCR but were still detectable using ddPCR (Figure 3A vs. Figure 3B and Figure 3C vs. Figure 3D).

## Discussion

In this study, we evaluated the performance of a ddPCR method in detecting *P. jirovecii* DNA in respiratory specimens. We first evaluated the analytical performance of the ddPCR method in detecting *P. jirovecii* DNA. A TaqMan primer and probe set was designed and the specificity of the set was verified (Table 3). The ddPCR assay was also subjected to additional validation and testing. The linear dynamic range of the assay was at least up to 10^5^ copies/test (test upper limit) of *P. jirovecii* DNA. The LoB and LoD of the assay was determined to be 0 and 3 copies/test, respectively (Table 4 and Figure 1). Then, we evaluated the clinical performance of this ddPCR method, testing the consistency of this method with qPCR in BALF and sputum. ddPCR and qPCR gave highly consistent results for BALF and sputum specimens. However, the detection rate of ddPCR was higher than that of qPCR in low-pathogen-load specimens, and all BALF specimens with inconsistent results were further tested by mNGS, which confirmed the ddPCR results. Testing of continuous respiratory tract specimens showed that the *P. jirovecii* load decreased after treatment, which implied that ddPCR was better in detecting low-pathogen-load specimens after treatment.

PCP is an opportunistic infection commonly seen in patients with HIV infection or other forms of immunosuppression. Non-HIV-infected patients at risk for PCP include patients with hematologic malignancies, solid tumors, or inflammatory disorders, and patients who have undergone bone marrow or solid-organ transplantation (28). Since *P. jirovecii* cannot be cultured, definitive diagnosis requires detection and identification of the organism by PCR assays of respiratory specimens, dye staining, or immunofluorescence staining, all of which may be influenced by the pathogen burden. For example, patients with HIV/AIDS have fewer neutrophils and a higher number of *P. jirovecii* in their sputum and BALF than other patients, making the diagnosis easier or more likely attainable. In contrast, patients with immunosuppression and without HIV infection likely have a low pathogen burden in their sputum and BALF, making the diagnosis difficult (29). Therefore, a highly sensitive nucleic acid detection method is required when the pathogen load is low.

qPCR is a common method to detect pathogen DNA in clinical laboratories, while ddPCR is an emerging method. The principles of these two methods are different, and therefore, the reagents and operations are not identical; for example, the number of amplification cycles is 40 in qPCR and 45 in ddPCR according to their respective instructions. Compared with qPCR, ddPCR is a more sensitive method used for the detection of low amounts of pathogens, including bacteria (30, 31), fungi (32, 33), and viruses (14, 34). In addition, ddPCR exhibits increased tolerance to interfering substances compared with qPCR (35–38). However, there was no study on *P. jirovecii* DNA detection using ddPCR, and our study supplies a new tool for PCP diagnosis. The results obtained here demonstrated that ddPCR had better sensitivity than qPCR in detecting *P. jirovecii* DNA. The majority of BALF samples (95.12%) showed coincident results by the two methods. The remaining four specimens were found positive by ddPCR but negative by qPCR, which were validated to be positive by mNGS. All these specimens had no more than 10 copies/test by ddPCR (Table 4), demonstrating the high sensitivity of ddPCR, especially in low-concentration specimens. However, in clinical practice, BALF is not easy to obtain because of the procedure's invasiveness, while sputum is easily obtainable. We found that 78.37% of the sputum specimens showed coincident results by the two methods, while eight specimens from six patients with PCP were positive by ddPCR but negative by qPCR (Table 5). These results highlight the possibility of missed detection of *P. jirovecii* DNA using qPCR, which may lead to delayed diagnosis of PCP.

Detection of *P. jirovecii* DNA can be used to monitor the therapeutic effects of PCP treatments (39–41), so we detected *P. jirovecii* DNA using qPCR before and during treatment (Figures 3A,C). In order to compare the detection rate, the same *P. jirovecii* DNA that has been detected by qPCR was also detected by ddPCR simultaneously (Figures 3B,D). After treatment, several specimens became negative according to the qPCR method. However, these specimens were positive by ddPCR, which implied that sulfonamides may still be necessary after two weeks of treatment. In particular, both sputum specimens from patient 6 were found negative by qPCR but positive by ddPCR, which likely led to the missed PCP cases when only qPCR was used. Thus, ddPCR is more sensitive than qPCR in detecting *P. jirovecii* DNA in both BALF and sputum.

In summary, we developed a ddPCR method for the detection and quantification of *P. jirovecii* DNA in the human airway. We evaluated its sensitivity for quantifying airway *P. jirovecii* load against a standard qPCR approach employing human airway specimens from patients with PCP. While both approaches can detect *P. jirovecii* DNA, ddPCR demonstrates better sensitivity for specimens with low abundance of the pathogen. Considering the importance of *P. jirovecii* detection in patients with immunodeficiency, ddPCR represents a useful, viable, and reliable alternative to qPCR in PCP patients.

## Data Availability Statement

The original contributions presented in the study are included in the article/supplementary material, further inquiries can be directed to the corresponding author/s.

## Ethics Statement

The studies involving human participants were reviewed and approved by the Ethics Committee of Peking Union Medical College Hospital (S-T767). Written informed consent for participation was not required for this study in accordance with the national legislation and the institutional requirements.

## Author Contributions

YX and YG conceived the study. JY and NW designed the study and wrote the manuscript. JY and XR conducted the experiments. JW, YT, JZ, and LZ contributed to data collection and data analysis. All authors read and approved the final manuscript.

## Funding

This work was supported by Beijing Gold-Bridge Project (No. ZZ21056), Beijing Key Clinical Specialty for Laboratory Medicine—Excellent Projects (No. ZK201000), and Tsinghua University Spring Breeze Fund (No. 2020Z99CFG010).

## Conflict of Interest

LZ and XR are employed by TargetingOne Corporation (Beijing, China). The remaining authors declare that the research was conducted in the absence of any commercial or financial relationships that could be construed as a potential conflict of interest.

## Publisher's Note

All claims expressed in this article are solely those of the authors and do not necessarily represent those of their affiliated organizations, or those of the publisher, the editors and the reviewers. Any product that may be evaluated in this article, or claim that may be made by its manufacturer, is not guaranteed or endorsed by the publisher.
