# Supplementary material for: Development of a Droplet Digital Polymerase Chain Reaction for Sensitive Detection of Pneumocystis jirovecii in Respiratory Tract Specimens
Source: Front Med (Lausanne). 2021 Dec 22;8:761788. doi: 10.3389/fmed.2021.761788 (PMC8727342; doi:10.3389/fmed.2021.761788)
Supplement: Supplementary file 1 [file Table_1.docx]

**SUPPLEMENTARY**

**TABLE. The continuous sampling dates and interval days of PCP patients**

| Patient number | Sampling date 1 | Sampling date 2 | Interval days |
| --- | --- | --- | --- |
| 1 | November 25, 2020 | December 2, 2020 | 7 |
| 2 | Feburary 1, 2020 | Feburary 7, 2020 | 6 |
| 3 | August 16, 2019 | August 22,2019 | 6 |
| 4 | August 1, 2019 | August 10, 2019 | 9 |
| 5 | November 13, 2020 | November 17, 2020 | 4 |
| 6 | Feburary 3, 2020 | Feburary 7, 2020 | 4 |
| 7 | September 8, 2020 | September 15, 2020 | 7 |
| 8 | September 25, 2020 | October 15,2020 | 20 |
| 9 | August 28, 2020 | September 5, 2020 | 9 |
